# Hypertension Care Coordination and Feasibility of Involving Female Community Health Volunteers in Hypertension Management in Kavre District, Nepal: A Qualitative Study

**DOI:** 10.5334/gh.872

**Published:** 2020-10-23

**Authors:** Jingru Tan, Hanzhang Xu, Qiping Fan, Olivia Neely, Rinchen Doma, Rishika Gundi, Binjwala Shrestha, Abha Shrestha, Shrinkhala Shrestha, Biraj Karmacharya, Wanbing Gu, Truls Østbye, Lijing L. Yan

**Affiliations:** 1 Global Health Research Center, Duke Kunshan University, Kunshan, Jiangsu, CN; 2 Department of Family Medicine and Community Health, Duke University School of Medicine, Duke University, North Carolina, US; 3 Duke University School of Nursing, Duke University School of Medicine, Duke University, North Carolina, US; 4 Department of Health Promotion and Community Health Sciences, Texas A&M University, Texas, US; 5 Duke Global Health Institute, Duke University, Durham, North Carolina, US; 6 Department of Community Medicine and Public Health, Teaching Hospital, Kathmandu, NP; 7 Department of Community Medicine, Kathmandu University School of Medical Sciences, NP; 8 Department of Public Health and Community Programs, Kathmandu University School of Medical Sciences, NP; 9 Vital Strategies, Beijing, CN

**Keywords:** hypertension management, community health workers, female community health volunteers, Nepal, qualitative research

## Abstract

**Background:**

Hypertension and related complications are major contributors to morbidity and mortality in Nepal. Community health workers have been proposed as promising health cadres to meet the growing healthcare demand for non-communicable disease management in other developing countries.

**Objective:**

We aimed to explore existing workflows, needs and challenges for hypertension care coordination and to assess the feasibility of establishing a Female Community Health Volunteer (FCHV)-based hypertension management program in Kavre, Nepal.

**Design:**

We conducted one focus group discussion with eight FCHVs and twenty-three in-depth interviews with four FCHVs not attending FGD, nine individuals with hypertension, six health workers, and four health officials in two village development committees of Kavre District, Nepal. Applied thematic analysis was performed using NVivo 12.

**Results:**

Health literacy related to hypertension was low among both community members and FCHVs. Delay in treatment initiation and loss to follow-up were common patterns despite anti-hypertensive medication compliance. Major health system-related barriers included underutilization of primary healthcare institutions, communication gaps and lack of grass-roots level educational campaigns. Community pharmacies, monthly health camps and increasing governmental attention to NCDs were favorable for improving hypertension management. This study also supports that FCHVs should be provided with adequate training and financial incentives to promote hypertension education, screening and referral in their catchments.

**Conclusions:**

Barriers and facilitators identified in this study provide important implications for future hypertension management in Nepal. We recommend hypertension education and screening across Nepal at a grass-root level through FCHVs. Providing professional training and proper financial incentives for FCHVs are warranted.

**Highlights:**

- Health literacy related to hypertension was low among both community members and Female Community Health Volunteers in Nepal.
- Delay in treatment initiation and loss to follow-up were common despite relatively high anti-hypertensive medication compliance.
- Health system-related barriers in research sites included underutilization of primary healthcare institutions, communication gaps, and lack of grass-roots level educational campaigns.
- Female Community Health Volunteers should be provided with adequate training and financial incentives to promote hypertension management.

## Introduction

Hypertension is a major modifiable risk factor for cardiovascular disease [1]. The prevalence of hypertension in Nepal has tripled in the last 25 years, currently reported to be between 22.4% and 38.6% [2,3]. The mortality rate of hypertension has also been steadily increasing in Nepal, from 135.6 to 145.2 per 100,000 persons from 1995 to 2015 [4]. Hypertension and related complications have become major contributors to deaths and disabilities in Nepal [5]. Although blood pressure can be lowered by simple interventions such as antihypertensive medication, hypertension control proportion is consistently reported to be low, ranging from 12% to 24% in Nepal [6,7]. This gap demonstrates that substantial disease burden can be averted through strengthening hypertension care. However, the health system in Nepal is challenged to meet the growing healthcare demand for non-communicable diseases (NCDs) due to a lack of human resources [8]. The Global Health Observatory data indicated that there were only 31 nurses and midwives per 10,000 population in 2018 – this constitutes more than 50% of the health workforce in Nepal [9].

Surveys in other developing countries have suggested community health workers (CHWs) can be promising health cadres for NCDs management [10,11,12]. There are around 51,500 CHWs, known as Female Community Health Volunteers (FCHVs) in Nepal, providing basic health services including family planning, maternal and child health care, and linking communities to government health centers [13]. At least one FCHV serves in each ward, the smallest local administrative body with roughly 150 households [13]. FCHVs usually receive 18-day basic training delivered by local health facility staff [14]. The national FCHV survey in Nepal shows the median age of FCHVs is 41.3 years, and 45% of them have completed primary education [15]. FCHVs has been found to be efficient to lead the home-based lifestyle intervention with regular monitoring to reduce blood pressure level in western Nepal [16]. However, the scalability and sustainability of FCHV-based hypertension management remain unknown, given the needs of the intervention population, FCHVs compensatory system, and little assessment for hypertension management coordination.

To further culturally appropriately incorporate FCHV into hypertension education, screening, and referral work in Nepal, more empirical evidence is needed to understand current practice, potential obstacles in hypertension management coordination and the needs of FCHVs for delivering hypertension related health services. In this study, we aimed to collect comprehensive insights from multiple stakeholders to understand their awareness of hypertension and its treatment and to assess the feasibility of FCHV participation in community-based hypertension management in Nepal.

## Methods

### Study setting

This qualitative study was conducted in two village development committees (VDCs) of the Kavrepalanchok (Kavre) District from June to July of 2018. We selected two VDCs to represent two primary tiers of the government health system and an outreach center as a not-for-profit community-based institution (Table 1).

**Table 1:** Characteristics of two study sites.


**Kavre District** Area: 1,396 km^2^ Population: 375,211 Wards: 135	**Dhunkharka VDC** Population: 8,121 No. of FCHV: 9 Health facility: a private primary health center Geographic characteristics: hilly area
	**Panchkhal VDC** Population: 14,930 No. of FCHV: 18 Health facility: a public primary health center Geographic characteristics: flat area


### Participants

We conducted a focus group discussion (FGD) with eight FCHVs to get a general idea of FHCVs’ role and responsibility and another four in-depth interviews (IDIs) with FCHVs to understand individual experiences. Additionally, we conducted in-depth interviews with nine individuals with hypertension, six health workers, and four health officials. The individuals with hypertension were recruited from the 168 participants identified by previous screening and completed quantitative survey questionnaires (these procedures are described elsewhere). We set a sampling quota before recruitment and interviewed three male and three female individuals with hypertension in two age groups (40–65, and 65+). We aimed to recruit equally representative participants with different duration of hypertension from three groups (less than five years, 5–10 years, and more than 10 years). Individuals with hypertension and FCHVs were recruited using convenience sampling until the themes reached saturation. Finally, nine individuals with hypertension were recruited due to time limits and saturation. All six health workers in the community health facilities and four health officials in selected VDCs were interviewed due to a limited number.

### Procedures

Selected individuals with hypertension were called and invited to participate in a home-based interview. All FCHVs, health workers, and policy makers were approached with the approval letter from Kavre District Office.

All IDIs and the FGD took place in a quiet and private location and were moderated in the local language (Nepali) by two local experienced research assistants. Both research assistants received half-day qualitative research training delivered by an experienced expert. Each IDI took 20–30 minutes to complete, while the FGD lasted 1.5 hours. All participants provided written informed consent before interviews. Interviews followed three semi-structured interview guides (Table 2) developed by the research team under the guidance of scholars from the Teaching Hospital (Nepal) and Duke University. All interview guides were first developed in English and then translated into Nepali. These guides consisted of opening questions and follow-up probes. Minor modifications to the interview guides were made during data collection based on feedback and emerging data. With consent from the participants, all interviews were audio-recorded. Immediately after interviews, there were debriefing sessions among all fieldwork members to summarize key information and limitations of the interview.

**Table 2:** Interview Contents for Each Group of Interviewees.

Interviewee category	Interview guide focus

**Individuals with hypertension**	Disease history Knowledge of hypertension Hypertension treatment and non-pharmaceutical management Treatment adherence Challenges of disease management
**FCHVs**	Current responsibility and role Knowledge and experiences about hypertension management Attitudes towards participating in hypertension management Perceived challenges and solutions
**Health workers & Health officials**	Available hypertension services in the local community Community awareness of hypertension Interaction with FCHVs Perception of FCHV mobilization Suggestion for FCHV mobilization


### Analysis

Interview recordings were transcribed verbatim and simultaneously translated into English by two bilingual research assistants. Thematic analysis was used to identify and organize themes [17]. The researchers read the transcripts several times to become familiar with the data. The codebook was developed in two phases. Firstly, the first author extracted some candidate codes from the interview guides and research questions. Then, two independent coders pre-coded three transcripts to revise and refine the codebook [18]. All transcripts were double coded through NVivo 12 software (average Kappa 0.704). Any discrepancies were discussed in the group until consensus was reached. If a consensus could not be reached, decisions were made by the first author.

### Ethical considerations

All study procedures are approved by the Institutional Review Board of Duke University and Nepal Health Research Council. Written informed consent was obtained from all participants. Study objectives and confidentiality issues were discussed with participants before interviews.

## Result

### Description of the sample

A total of 23 IDIs and 1 FGD were conducted. The mean ages of individuals with hypertension and FCHVs were 64.4 (±13.3) and 47.6 (±8.9), respectively. The mean hypertension duration of individuals with hypertension was 6.4 (±4.4) years. Two-thirds of individuals with hypertension reported one or more comorbidities. None of the individuals with hypertension were insured, as the national health insurance rate is only 5% in Nepal [13]. FCHVs’ mean years of working was 20.3 (±8.7) years. The demographic characteristics of all interviewees were presented in Table 3. Based on three domains: hypertension treatment and awareness, health system-related barriers and facilitators of hypertension care utilization, and feasibility of FCHV mobilization, Tables 4, 5, 6 summarize key themes and findings by domain.

**Table 3:** Demographic Characteristics of all Interviewees.

Characteristics (Mean ± SD or %)	Individuals with Hypertension (n = 9)	FCHVs (n = 12)	Health Workers (n = 6)	Health Officials(n = 4)

**Age (years)**	64.4 ± 13.3	47.6 ± 8.9	–	–
**Female (%)**	66.7	100.0	16.7	0
**Comorbidities (%)**	66.7			
**HTN duration(years)**	6.4 ± 4.4	–		
**Working (years)**	–	20.3 ± 8.7	–	–
**Highest education level**				
illiterate	66.7	41.7	–	–
Primary school	22.2	41.7	–	–
Secondary school	0	16.6	–	–
Higher	11.1	0	–	–
**Occupation**	4 farmers 1 teacher 1 retired 3 None	– – – –	1 nursing midwife 2 doctors 3 health assistants	1 ward executive 1 senior auxiliary health worker 1 FCHV focal person 1 public health officer


**Table 4:** Hypertension awareness and treatment.

Key themes	Major findings

**Knowledge of hypertension**	Most individuals with hypertension could link hypertension to its causes, symptoms and complications. Misconceptions and home remedies were prevalent among individuals with hypertension.
**Hypertension treatment initiation**	There was a delay in treatment initiation. Negligence, distance to health institutions and fear of lifelong medication-taking were main barriers to treatment initiation.
**Medication adherence**	Overall, individuals with hypertension adhered to medication once they started it Some individuals with hypertension occasionally stopped medication due to forgetfulness, negligence, laziness, and affordability issues
**Regular checkups**	Loss to follow-up was common among individuals with hypertension
**Non-pharmaceutical management**	Individuals with hypertension complained it was hard to regulate their lifestyle Family support was an important part of regulating a healthy lifestyle.


**Table 5:** Health system-related barriers and facilitators of hypertension care utilization.

Key themes	Major findings

**Health system-related barriers**	Underutilization of primary healthcare institutions, communication gap and lack of grass-roots level educational campaigns were main challenges.
**Health system-related facilitators**	Community pharmacies and health camps increase hypertension care accessibility Recent NCDs-related actions were favorable for hypertension management improvement.


**Table 6:** Feasibility of FCHV mobilization.

Key themes	Major findings

**The readiness of FCHV-based hypertension educational, screening and referral campaigns**	FCHVs’ responsibility had been expanding to lifestyle education and counselling gradually All stakeholders had a positive attitude to FCHV mobilization
**Prerequisites of FCHV-based hypertension educational, screening and referral campaigns**	Systematic training, appropriate incentives and adequate access to equipment and medical resource were prerequisites of effective FCHV mobilization


### Hypertension awareness and treatment

#### Theme 1: Knowledge of hypertension

Most health workers and officials at both sites believed community members could link hypertension to some symptoms like headache, dizziness, eye pain and paralysis, but knew little about risk factors and specific complications of hypertension.

“Most people in this community have not been able to connect these things (heart disease, stroke and hypertension)”. (Health worker 05)

Individuals with hypertension demonstrated fundamental hypertension-related knowledge. All of them knew individuals with hypertension should reduce the intake of salt and fat and take exercise frequently. Half of them associated stress with hypertension.

“Hypertension, before, I heard that alcoholics get it. That… when the blood pressure’s high, head starts hurting and starts feeling dizzy. Something like that happens. No power…that happens. It affects the heart the most. Head also hurts. There are many diseases that this invites, according to the doctor. Paralysis happens”. (Male patient, 56, HTN 10 years)

Many health workers indicated there were confusion and misconceptions regarding hypertension in the community. Individuals with hypertension and FCHVs shared some home remedies and unusual practices for hypertension treatment, such as eating bitter plants, wearing acupressure slippers, and walking on the grass. The ward executive mentioned witch doctors still practiced in Tamang communities.

#### Theme 2: Treatment initiation

Most health workers and health officials mentioned individuals with hypertension didn’t seek treatment at an early stage. They said people in rural areas rarely go to a health institution unless health problems became a hindrance in their daily life.

“Until people have symptomatic issues like headaches, weird sensation in their hands and legs, pain in the eyes, only then people feel it as a necessity to visit the health center”. (Health worker 01)

Health providers suggested that the fear of lifelong medicine use was the strongest reason behind this delay. Another obstacle was the long transportation distance from the patient’s home to the health institutions, which was partially due to the landscape geography in Nepal. Individuals with hypertension didn’t raise the economic burden as a major concern.

“People have the fear of hypertension and diabetes. They know that things will get difficult when they have these diseases”. (Health worker 05)

“People might have to walk for 2–6 hours to reach the health institution. Since the health institutions are far, people think why they should go to those health institutions for minor cases”. (Health official 04)

#### Theme 3: Medication adherence

Most individuals with hypertension had no difficulties following their medication regimen. They simply said that “It’s already a habit”. Five of them reported they had occasionally stopped taking medication due to forgetfulness, negligence, laziness, and affordability issues. Two reported medication cessations when they felt better, but when their blood pressure rose again, they realized the importance of taking medication daily.

I stopped taking medicine for 1 year. And again, I felt ill and had to continue medicine. (Female patient, 59, HTN 5 years)

Overall, individuals with hypertension adhere to the medication once they start it.

When they come for follow-ups, at most, they would have lessened the dosages, but almost all of them continue their medication. (Health worker 04)

#### Theme 4: Regular checkups

Regarding regular blood pressure monitoring, few individuals with hypertension had weekly checkups, according to health workers and nurses’ reports. One health worker in Panchkhal revealed, “They take their medication but do not come for follow-up”. Individuals with hypertension conveyed their perceptions of little importance of blood pressure measurement: “blood pressure is fine, why should I go?”.

#### Theme 5: Self-management of hypertension with family support

All individuals with hypertension conveyed the difficulty of strictly regulating their lifestyle. The major disruption to their healthy behaviors included burdens of a busy life, oily and salty dietary habits, and comorbidities such as asthma and COPD that prevented them from being physically active. Alcohol abuse was commonly observed among the Newar group, increasing the difficulty for them to self-manage their blood pressure. Several individuals with hypertension were also frustrated with little subjective improvement despite long-term efforts. One patient said that “even though I did that much … I don’t know … I didn’t feel better” (Male patient, 85, HTN 1.5 years).

Family support was a regulatory aspect of a healthy lifestyle. Most individuals with hypertension said their family didn’t cook food separately for them, but the manner in which the food was cooked and the use of ingredients were changed to suit a hypertensive person’s diet. Family members also helped purchase medicine, reminded individuals with hypertension to take medicine on time, accompanied individuals with hypertension to appointments, and provided transportation when they needed to go to health facilities.

### Barriers and facilitators of hypertension care utilization

#### Theme 1: Health system-related barriers

There was an inconsistency regarding the reported accessibility to hypertension care between health providers and individuals with hypertension. Most health workers and health officials in Dhunkharkha believed people had “very easy access” to primary health institutions, with secondary and tertiary hospitals being “not very far to reach”.

However, nearly all individuals with hypertension reported they only went to hospitals for treatment or checkups. One patient is Dhunkharha complained:

“There aren’t any physician doctors or consultants. I need to go outside for them. There is only the normal, measuring pressure. Special or particular services aren’t here”. (Male patient, 42, HTN 2 years)

Also, according to the “Free essential health services”, all drugs provided in HPs or PHCs should be free of charge to all individuals, which was confirmed by health workers. “All of the medications available here are free of cost … For hypertension, we have here beta-blockers (propanol), furosemide, calcium channel blockers (lacidipine, amlodipine)”. However, none of the individuals with hypertension reported that he/she had gotten any free hypertensive drug from the PHC.

Conversations with health workers and individuals with hypertension demonstrated some communication gaps. One FCHV suggested a few individuals with hypertension were unaware of “Free essential health services”.

“If they ask us where they can get medicine then we take them to PHC, they said it take charge … and we told them it didn’t take any charge. and we gave them that suggestion and then they come here”. (FCHV, 51, working 30 years)

When one health official reported, “we are regularly organizing health camps and spreading information”, several individuals with hypertension didn’t think they had been informed of such activities.

Hence, health workers indicated that grass-roots level informational awareness campaigns addressing the consequences and complications of hypertension, as well as services offered by local health institutions, were highly needed in Nepali communities. One executive believed the most effective awareness interventions should educate women and children above grade 5 about hypertension, noting:

“If we were to spread awareness about anything, then women and children studying in grade 5 or above would be the best path towards it. If we were to include children from grade 5–10, then we could inform many people. These children will absorb the information quickly”. (Health official 01)

One health worker offered the idea that educational programming should specifically target high-risk populations over 40 years old and those who had a family history of hypertension. Multiple health workers recommended that residential message boards, dramas, radio and TV programming were effective ways to deliver health education.

#### Theme 2: Health system-related facilitators

Community pharmacies, run by non-professional pharmacists with basic orientation training on pharmacology and drug dispensing, were mentioned by six interviewees as another place to seek health services in both sites. Some of these community pharmacies also provided blood pressure measurement services. One patient said:

“It takes 5 minutes on foot to reach the ‘Khaba medical store’. It takes about 2 hours on bus to reach hospital from home”. (Female patient, 59, HTN 5 years)

Monthly mobile health camps, which aim to provide healthcare to communities lacking medical resources, facilitated hypertension diagnoses in remote areas. Some health workers mentioned hypertension-related knowledge had been spread in recent health camps.

In conversations, health workers and health officials indicated that NCD management has been receiving increased attention and resources. Several NCD-related programs had been piloted in other districts. The most important one was the “package of essential noncommunicable diseases (PEN)”, which aims to provide free preventive measures, consultations and referrals for NCDs (for which hypertension is an important risk factor). This program was expected to be scaled up across the country in the coming years.

### Feasibility of FCHV mobilization

#### Theme 1: Readiness for FCHV-based hypertension educational and screening campaigns

Although traditionally, FCHVs’ responsibilities were focused on maternal and child health, their work has been gradually expanded to some forms of hypertension management, and they have been familiar with medication reminder work. FCHVs have started to receive basic hypertension-related information from meetings and training and spread it to the community. One FCHV said, “we have heard it little bit from sir in PHC during training, but we have not heard about it detailly”. Two FCHVs mentioned they had regularly checked in with TB patients to ensure that they were taking medication daily. These procedures shared similarities with the reminder-work that would be required in future work for hypertension management.

All health workers and health officials had confidence in FCHVs’ potential to take the responsibility of community-level hypertension management. They considered FCHV mobilization for hypertension could be a “fruitful endeavor”.

“Yes, I think they can do it. They are in regular contact with the people of their area. If they identify someone with high BP, they can go to their house and measure their BP for 4–5 days, even if the patients themselves are physically incapable of doing so”. (Health worker 06)

FCHVs also showed a positive attitude toward participating in hypertension management. They felt self-fulfillment and were motivated to “know about the new things” and “give more suggestions to the community people”.

#### Theme 2: Prerequisites of FCHV FCHV-based hypertension educational and screening campaigns

Adequate training was frequently mentioned by most interviewees as the cornerstone of the success of an FCHV-based hypertension management program. FCHVs said, “we cannot consult blood pressure patients, we need training, then we can do that”.

Training courses ought to be appropriately designed for low-literacy health workers, given one-third of the FCHVs were senior citizens, and half could not read or write. One health official stated that previous FCHVs’ reporting format was based on markings. “For example, if they have provided ORS, there is a picture of the ORS on the report. They just mark on the picture.”

All health workers and health officials suggested that financial motivation would be a significant prerequisite for future FCHV mobilization. As volunteers, FCHVs didn’t receive regular salaries, but only small incentives from the government. Although promised to be given Rs.1000 (USD 8.93) per month from 2018, one FCHV revealed “we didn’t get till now I don’t know they will give or not”. She hoped that the government would establish the role of FCHV as a permanent job with a regular salary and pension after retirement.

Additionally, FCHVs indicated the difficulties of working without the necessary equipment from organizations. One FCHV said, “We have to walk to far places even in heavy rainfall, but umbrella is not provided to us. We also have to walk in evening but there is no any organization that has provided us a torch light”. A lack of sufficient basic medicines and health facilities were also mentioned as challenges for FCHVs’ work.

## Discussion

This study explored barriers and facilitators in hypertension treatment and control perceived by individuals with hypertension, healthcare providers and policy makers, and provided a comprehensive hypertension management scenario. Furthermore, it assessed the readiness and feasibility of FCHV-based hypertension management programming. Hypertension awareness was low. Delay in treatment initiation and loss to follow up were common patterns among hypertensive patients despite compliance with medication. Underutilization of primary healthcare institutions, communication gaps and lack of grass-roots level educational campaigns were identified as major health system-level barriers. Community pharmacies, monthly health camps and increasing governmental attention to NCDs were favorable for hypertension management. This study also showed that FCHVs had the potential to promote hypertension education, screen for hypertension and refer in their catchments, with adequate training and proper motivation.

There was poor knowledge of hypertension among the community, delay in treatment initiation and lost to follow up were the main challenges for blood pressure control. Low health literacy and negligence were serious challenges. Previous studies from the U.S. suggested that individual healthcare utilization had a positive association with health literacy [19,20]. Patients unable to connect elevated blood pressure with specific negative outcomes showed more negligence to hypertension and consequently were less likely to start treatment promptly. Besides, asymptomatic patients barely sought healthcare due to the traditional perception of symptoms-based treatment [21,22]. Misconceptions like wearing slippers, adding water to yogurt and salty food, and trust in witch doctors, as cited in another study, interfered with blood pressure control [22]. Additionally, the individuals with hypertension in this study reported they were reluctant to start pharmaceutical treatment due to fears associated with the long-term nature of hypertension medication, which is congruent with Sachita et al.’s research [22]. Few individuals with hypertension in this study raised financial hardships as obstacles to treatment decisions. One possible explanation is that individuals with hypertension who completely cannot afford the treatment were excluded by the criteria “under medication”. In addition to the negligence of checkups, individuals with hypertension were often older adults who suffered from multiple comorbidities requiring more comprehensive examination and consultation than what is available in primary health institutions in resources-constrained countries like Nepal. Hence, patients rarely went back to primary health centers for follow-ups and community health centers normally lost the track of patients after referral. The capacity of primary health institutions in hypertension management was not fully functional. Distance to hospitals and transportation barriers further deterred participants from regular checkups. Overall, these findings demonstrated the necessity to promote community-based hypertension education programs, promote early treatment, and set up an effective downward referral system.

Our study found insufficient communication and a lack of transparency between individuals with hypertension and the health providers, which led to the underutilization of primary health institutions. As supported by previous studies, patient-doctor communication is integral to ensure trust and proper management of health [23]. This emphasizes the importance for healthcare providers need to increase their awareness of the needs in their community. Additionally, more efforts need to be made to make the community aware of the prevalent services which are already available. The aforementioned community-based education campaigns should also be responsible for communicating available health services in primary health institutions. Interview with individuals with hypertension showed that community pharmacies were popular places for anti-hypertension drug purchases, given the convenience and accessibility compared to health centers or hospitals that are further and have long waiting times [6]. There are approximately 20,500 officially registered pharmacies across Nepal in both urban and rural areas [13]. Community pharmacies are normally operated by “lay persons” who receive training that ranges from a few weeks to three months [24]. If further NCD-related training and steady drug supply are provided to the pharmacy operators, there can be an improved quality of service for people who frequently visit pharmacies for a basic consultation.

There is a great potential in designing FCHV-based educational, screening and referral programs for hypertension to be implemented at the grass-roots level. FCHVs were identified to be effective candidates in delivering hypertension care because of their trustworthiness among community members, health workers, and health officials, confirmed by other studies in Iran and South Africa [25,26]. Our study found that FCHVs had limited knowledge of hypertension, but some familiarity with ‘reminder work’ that would be required for HTN management such as reminding individuals with hypertension to take medication. Experience from India sets a template for designing a stepwise training and effective supervision mechanism for CHWs with little formal education in hypertension screening and management [27]. Similar to the findings of Neupane et al., despite having very limited knowledge about hypertension, FCHVs in our study were enthusiastic about learning more and confident in their ability to manage HTN in the community with proper training [28]. While FCHVs were eager to learn more about HTN management, they stressed the importance of external motivators and compensation in order to sustain a community-based hypertension intervention. Singh et al.’s study also suggests well-trained community health workers who receive regular payment are more likely to engage the community in grass-roots health-related empowerment [29]. A justifiable salary system should be developed for FCHV mobilization to define payer and payment mechanism. Having access to basic equipment such as digital blood pressure monitors and antihypertensive drugs would also improve FCHVs’ performance. FCHVs empowerment should also be considered as an indispensable component for extension of PEN program. The PEN program was introduced to Nepal in 2016 to reduce the burden of NCDs. It consists of a set of cost-effective interventions, such as lifestyle modifications, early detection, and low-technology diagnosis, timely referral and affordable medicines, that can be delivered at primary health centers. Although the providers of the PEN program in Nepal have been currently limited to primary official health staff, FCHVs have great potential for managing relatively simple tasks like health promotion, basic lifestyle counselling, blood pressure measurement, and referral of patients with suspected hypertension.

## Strengths and limitations

This was the first qualitative study in Nepal involving a range of stakeholders to gather multidimensional insights into hypertension management – previous studies of this issue have only involved patients or health providers. Although the qualitative design and small sample size limit the generalizability of the study findings, we intentionally selected two sites with different economic and geographic characteristics to cover a wide range of patient characteristics. Furthermore, we included all health providers, health officials and two-thirds of FCHVs from the study sites and used a sampling quota to reduce selection bias.

## Conclusion

Lack of awareness, delay in treatment initiation, and loss of follow-up are major challenges in hypertension treatment and control in rural Nepal. Grass-root level hypertension screening and education campaigns can be effective strategies to improve timely healthcare utilization. Once they have been empowered with appropriate training and motivated by proper incentives, FCHVs promise health cadres for the promotion of hypertension education, screening and referral in their catchment areas.
